# MiRNA‐199a‐5p targets WNT2 to regulate depression through the CREB/BDNF signaling in hippocampal neuron

**DOI:** 10.1002/brb3.2107

**Published:** 2021-08-01

**Authors:** Zheng Liu, Jianli Yang, Qing Fang, Hua Shao, Dalu Yang, Junfang Sun, Lizhi Gao

**Affiliations:** ^1^ Department of Graduate School Tianjin Medical University Tianjin China; ^2^ Department of department of neurology The Second Affiliated Hospital of Baotou Medical College Baotou China; ^3^ Department of Clinical Psychology Tianjin Medical University General Hospital Tianjin China

**Keywords:** CREB/BDNF, depression, miR‐199a‐5p, WNT2

## Abstract

**Introduction:**

This study mainly investigated the role of miR‐199a‐5p in depression.

**Methods:**

qRT‐PCR and western blotting were employed to detect the expressions of miR‐199a‐5p, CREB and BDNF. Sucrose preference test, forced swimming test, and tail suspension test were performed to evaluate depression‐related symptoms. MTT assays and flow cytometry were used to examine the cell reproduction and apoptotic cells of hippocampal neuron.

**Results:**

The data demonstrated that the expression levels of miR‐199a‐5p in the cerebrospinal fluids and serums of depression patient and the hippocampus of chronic unpredictable mild stress (CUMS) mouse were significantly increased. However, the expressions of WNT2, p‐CREB, and BDNF were inhibited. In addition, miR‐199a‐5p‐inhibitor enhanced sucrose preferences of CUMS mouse and decreased immobile time in sucrose preference test and forced swimming test. Knockdown of WNT2 attenuated the effects of miR‐199a‐5p‐inhibitor on cell reproduction and apoptotic cells of hippocampal neuron and the expression of WNT2, p‐CREB, and BDNF.

**Conclusion:**

MiR‐199a‐5p can target WNT2 to enhance the development of depression through regulation of the CREB/BDNF signaling. Trial registration: JNU‐Hos‐49284.

## Graphical abstract

miR‐199a‐5p can target WNT2 to enhance the developments of depressions through the regulations of CREB/BDNF signaling.

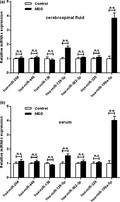

## 1 INTRODUCTION

Depression is one of the major public health concerns, and chronic depression may cause neuron and synapse atrophies in the limbic system (Moussavi et al., 2007; Strine et al., 2009). The pathology of depression is related with complex interactions among genes, immune‐stimulated inflammations, as well as environmental factors. It is well‐established that neuron plasticity might be changed in some important fields in depressive brain (Castren, 2013). The changes of gene expression detected in blood and dermal cells suggested that genetic defects may have susceptibility for the induction of major depression disorders (Roy et al., 2017; Y. Xu et al., 2010).

MicroRNA (miRNA) is a family of small noncoding RNAs (18–22 nt) that can regulate the expression of target messenger RNAs through the interference with transcription or inhibiting translation (Dwivedi, 2014). Studies have revealed that miRNAs play critical roles in various cellular biological procedures, such as metabolism, cell survival, differentiation and apoptosis (Wan et al., 2015). Dysregulation of specific miRNAs may contribute to various human diseases, including cancer, Alzheimer's disease, and Parkinson's disease (Martins et al., 2011). Studies have identified miRNAs that are involved in depression. For example, miR‐1202 was reported to be a primate‐specific and brain‐enriched miRNA involved in major depression and antidepressant treatment (Lopez et al., 2014). MiR‐199a‐5p plays a suppressive role in tumor cell reproduction (Raimondi et al., 2014; H. Yi et al., 2013). This study was carried out to investigate the role of miR‐199a‐5p in the pathophysiology of depression.

The WNT pathway actively participates in embryogenesis, development of nervous system, and adult hippocampal neurogenesis (Huelsken & Behrens, 2002). The WNT signaling pathway was also demonstrated to play essential roles in the treatment of major depression. It was reported that WNT and its downstream factors were involved in the pathophysiology of depression (Voleti & Duman, 2012). In addition, elevated temporal cortex CREB concentrations in major depression were observed (Dowlatshahi et al., 1998). Another study reported that inhibited function of the CREB1/BDNF/NTRK2 pathway had a negative impact on the risk mechanism of depression (Juhasz et al., 2011). In this study, interactions among miR‐199a‐5p, the WNT pathway, and the CREB1/BDNF axis in the development of depression were investigated.

## 2 METHODS

### 2.1 Patients information and sample preparation

This study recruited MDD patient with standards of 1) Patients had major depressions diagnosed by Chinese Classification and diagnostic criteria of Mental Disorders, 3rd edition; 2) Patients had Hamilton Depression Scale scores >= 24; Patient were excluded if they had: 1) Primary organic diseases, mental illnesses, drug use, or history of bipolar disorders. The control group had no mental/neurological illnesses. This study was approved by the Ethics Committee of The second affiliated hospital of Baotou medical college. All participants signed the written informed consent.

Cerebrospinal fluid (CSF, 4 ml) was collected by lumbar puncture in 3 male MDD patients and 4 female MDD patients aged at 31.26 ± 8.05 years old, as well as 3 male control participants and 4 female participants aged at 32.38 ± 8.56 years old. Fasting venous blood samples (4 ml) were obtained in another 20 MDD patients (9 males and 11 females, aged at 34.84 ± 9.59 years old), and 20 healthy controls (10 males and 10 females, aged at 35.12 ± 10.06 years old). After collection, CSF and blood samples were centrifuged at 4°C for 10 min and then stored at −80°C.

### 2.2 Animal information

Adult male Kunming mice were purchased from the Vital River Laboratory Animal Technology Co, Ltd (Beijing, China). Mice were kept at 12 hr/12 hr day/night cycle and with free access to food and drink. Twelve mice were grouped into chronic unpredictable mild stress (CUMS) and control (*n* = 6) groups. Twenty‐four mice were grouped into the control, CUMS, CUMS + NC‐inhibitor, and CUMS + miR‐199a‐5p‐inhibitor (*n* = 6) groups. Another 18 mice were grouped into CUMS, CUMS + fluoxetine, and CUMS + fluoxetine +miR‐199a‐5p mimics (*n* = 6) groups. MiR‐199a‐5p‐inhibitor, miR‐199a‐5p mimic, and the control (Shanghai, China) were intracerebroventricularly administered to mice with 0.5 nmol/mouse (L.‐T. Yi et al., 2014). The injection was carried out one time per week for 3 weeks. The fluoxetine group had 10 mg/kg fluoxetine by intraperitoneal injections one time per day for 2 weeks. For euthanasia, animals were deeply anesthetized with sodium pentobarbital (30 mg/kg body weight, Sigma Chemical Co.) through intraperitoneal injection. Mice were finally sacrificed by cervical dislocation. All animal experiments were performed following the Institutional Animal Care Committee of The second affiliated hospital of Baotou medical college.

### 2.3 CUMS

Mice were subjected to long‐term mild stressors of: 1) Clipping the distal 1/3 mouse tail for a minute; 2) Foot shocking for 10 s; 3) Swimming at 4°C for 5 min; (4) Illuminations for overnight; 5) Fasten for 1 d; 6) Water deprivations for 1 d; 7) Wet cage for 1 d; 8) A 45 degree cage tilt for 1 d. Mice had one to two stressors every day for 42 d. The control mice had no stressor. After that, mice were subjected to sugar preference test, forced swimming test, and tail suspension test.

### 2.4 Sucrose preference test

After CUMS, mice were deprived of water after 8 p.m. The next day, mice had 1% sucrose with 1 hr/day for 3 d from 8 to 9 a.m. On the test day, sucrose preference test was carried out after 8 hr of water deprivation. After that, mice had free access to drink 1% sucrose or water for 1 hr. The test last for 2 d, followed by switching of drink bottles. After the test, the weight loss of bottles was considered as liquid consumptions.

### 2.5 Forced swimming test

A glass beaker was filled with 18 cm of water at 22°C, and mice were forced to swim in it for 6 min. The immobility duration of mice was measured by double‐blinded observers, as the time that mice didn't move or had little movement. After forced swimming test, mice were cleaned and put back into the cage.

### 2.6 Tail suspension test

Mice were stuck by rubber at 1 cm to tail tip and suspended from top for six min. Immobility durations that mice were utterly motionless were calculated by double‐blinded observers.

### 2.7 Hippocampus

Mice were sacrificed after CUMS. The hippocampus was taken, frozen, and stored at −80°C. ELISA kits were used to detect the concentrations of corticosterone (CORT) in mouse serum.

### 2.8 QRT‐PCR

Total RNAs were isolated from tissues and cells using Trizol reagent (Invitrogen). cDNA samples were synthesized with reverse‐transcription and used as template for qRT‐PCR. U6 was used as the internal control. The expression levels were determined using 2^−ΔΔCt^ method. The primer sequences were listed in Table 1.

**TABLE 1:** Sequences of primers used in qRT‐PCR

Gene	Forward primer (5’−3’)	Reversed primer (5’−3’)
miR−199a−5p	GTCGATACCAGTGCGTGTCGTCCTGTCGGC	AATTGCACTGGATACGACAGCCTAT
U6	GCTTCGGCAGCACATATACTAAAAT	CGCTTCACGAATTTGCGTGTCAT

### 2.9 Western blotting

Hippocampus homogenate was lysed, centrifuged, and separated via SDS‐PAGE. Total proteins were transferred to PVDF membrane (Amersham). The membrane was blocked in 5% skim milk at 25°C for 1 hr, and then treated with anti‐WNT2, anti‐CREB, anti‐p‐CREB, anti‐BDNF, and anti‐β‐actin (Santa Cruz Biotechnology, US) at 4°C for overnight. Then, the membrane was treated with horseradish peroxidase‐labeled secondary antibody (Invitrogen, US). Chemiluminescence kit was used to visualize the signals (Biobyt, UK).

### 2.10 Cell culture and transfections

C57BL/6J newborn (P0) mice were obtained from Animal Center of The second affiliated hospital of Baotou medical college. Hippocampal neuron was taken from newborn mice and incubated in neurobasal medium with L‐glutamine and B27 (Life Technologies). MiR‐199a‐5p‐mimic, miR‐199a‐5p‐inhibitor or negative control (NC), and si‐WNT2 or si‐NC were transfected to hippocampal neuron using Lipofectamine 2000 (Invitrogen). Hippocampal neuron was analyzed after 1 d.

### 2.11 MTT assays

Hippocampal neuron was seeded in 96‐well plates in medium with 20 μl 5 mg/ml MTT and maintained at 37°C with 5% CO_2_for 4 hr. Next, the supernatant was removed and 150 μl DMSO was added. ELISA was used to measure the signals at 490 nm.

### 2.12 Flow cytometry

The hippocampal neuron was centrifuged at 600 rpm for 2 min. The supernatant was removed and 10 μl Annexin V‐FITC and 5 μl Propidium iodide were added for incubation at 4°C for 30 min in dark. The apoptotic cells were detected using a flow cytometer (Coulter).

### 2.13 Cell transfections

HEK293 cells were obtained from Gefan Biotechnology, China and maintained in DMEM with 10% FBS, 100 mg/L penicillin, and 100 mg/L streptomycin. Cells were cultured at 37°C with 5% CO_2_.

### 2.14 Dual‐luciferase reporter assay

The 3’‐UTR of WNT2 with the binding sequence of miR‐199a‐5p was amplified and cloned into PGL3 vector (Promega). WNT2‐WT (wild‐type) or WNT2‐MUT (mutant) was transfected with miR‐199a‐5p‐mimic/inhibitor to HEK293 cell. After 2 d, HEK293 cells were lysed. Luciferase activities were measured using Dual‐luciferase reporter assay (Promega, US).

### 2.15 Statistical analysis

SPSS 17.0 was used for data analysis. The data were expressed as means ± standard deviation (*SD*). For each analysis, the data sets were firstly analyzed for their normality (Shapiro–Wilk test) and equal variance (modified Levene test) assumptions prior to the use of parametric statistical methods. Then, differences between 2 groups were evaluated by Student's *t* test, and difference in multiple groups was evaluated by one‐way analysis of variance (ANOVA) followed by post hoc analyses test if data conformed to normality and homogeneity of variance. *p* < .05 was considered statistically significant.

## 3 RESULTS

### 3.1 The expression levels of miR‐199a‐5p were significantly elevated in the serum of CSF and MDD patient

**FIGURE 1:**
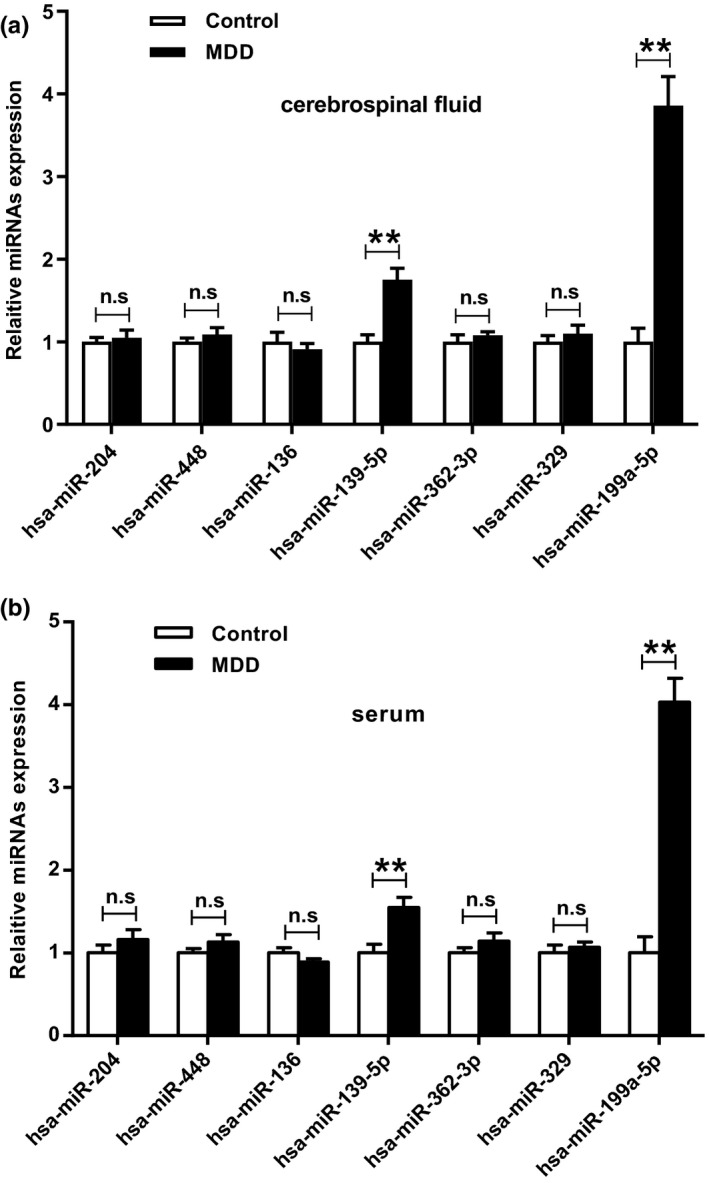
The expression of miR‐199a‐5p in MDD patient. (a) The expression of seven miRNAs for CSF from MDD patient. (b) The expressions of seven mRNAs in serum of MDD patient. **p < .01

The expression of miR‐139‐5p and miR‐199a‐5p were detected in CSF and serum of MDD patient. It showed that the expression levels of miR‐139‐5p (*t* test, t_(4)_ = −8.015, *p* = .001) and miR‐199a‐5p (*t* test, t_(4)_ = −12.856, *p* =.000) were elevated in CSF, while the elevation of the expression levels of miR‐199a‐5p were more obvious, compared with the control patient (*p* < .01) (Figure 1a). The same trend was observed in MDD serum (miR‐139‐5p, *t* test, t_(4)_ = −6.057, *p* = .004; miR‐199a‐5p, *t* test, t_(4)_ = −15.119, *p* = .000) (*p* < .01) (Figure 1b).

### 3.2 The expression of MIR‐199a‐5p in the hippocampus of cums mice

**FIGURE 2:**
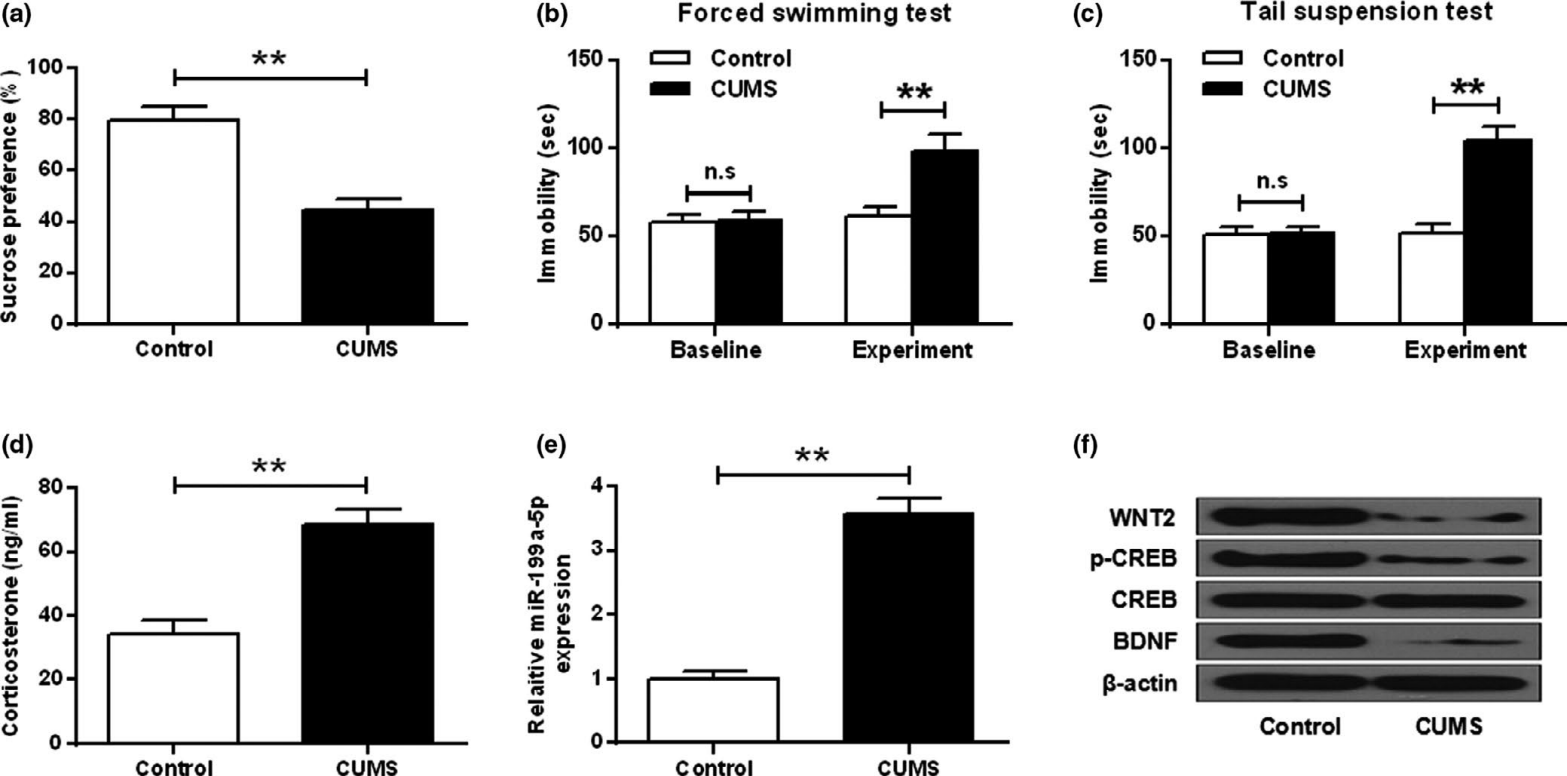
The expression of miR‐199a‐5p in CUMS hippocampus. (a) sucrose preference test. (b) forced swimming test. (c) tail suspension test. (d) CORT concentrations in CUMS serum. (e) Expression of miR‐199a‐5p in CUMS hippocampus. (f) The expression of WNT2, p‐CREB, and BDNF in CUMS hippocampus. **p < .01

A great reduction of sucrose preference in CUMS was observed (*t* test, t_(10)_ = 8.648, *p* = .001) (*p* < .01) (Figure 2a). And the immobility duration in the forced swimming test (ANOVA, *F*_(3,20)_ = 26.138, *p* = .000; LSD test, t = −6.891, *p* = .000) (Figure 2b) and tail suspension test (ANOVA, *F*_(3,20)_ = 68.640, *p* = .000; LSD test, t = −11.617, *p* = .000) (Figure 2c) was significantly longer than that in the control (*p* < .01). In addition, the concentration of CORT in CUMS serum was significantly enhanced (*t* test, t_(10)_ = −9.086, *p* = .001) (*p* < .01) (Figure 2d). As shown in Figure 2e, the expression levels of miR‐199a‐5p in CUMS mice were significantly elevated compared to that in the control mice (*t* test, t_(10)_ = −16.274, *p* = .000) (*p* < .01). Western blotting results demonstrated that the expression of WNT2, p‐CREB, and BDNF in CUMS mice were remarkably reduced compared to that in the control (*p* < .01) (Figure 2f).

### 3.3 MIR‐199a‐5p suppressed cell reproduction and enhanced apoptotic cells of hippocampal neuron

**FIGURE 3:**
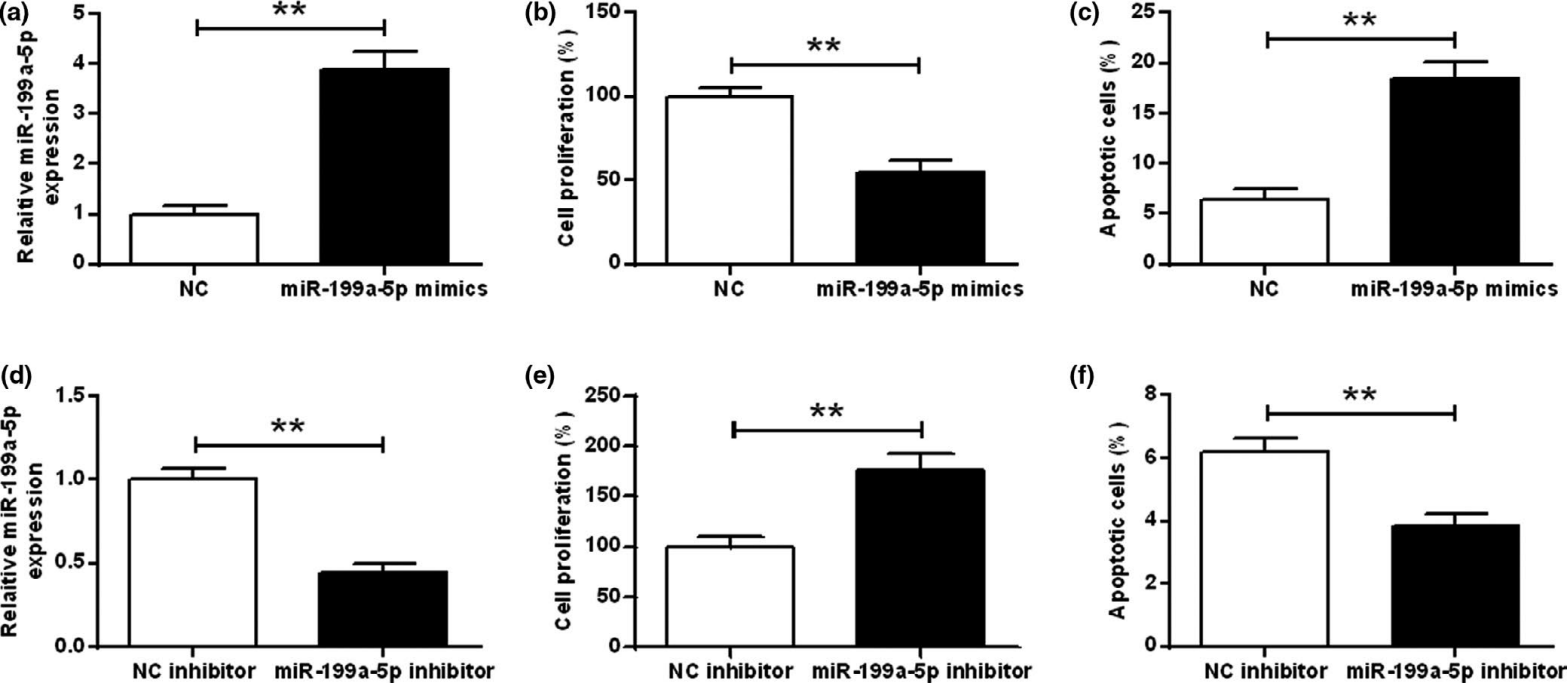
Effects of miR‐199a‐5p on the cell reproduction and apoptosis of hippocampal neuron in mice. Hippocampal neuron of neonatal mice was transfected by miR‐199a‐5p mimics or‐inhibitor. (a and d) The expression of miR‐199a‐5p. (b and e) The cell reproduction of hippocampal neuron. (c and f) The apoptosis of hippocampal neuron. **p < .01

To explore the role of miR‐199a‐5p, hippocampal neuron was transfected with miR‐199a‐5p mimic or miR‐199a‐5p‐inhibitor. It was found that miR‐199a‐5p mimic promoted the expression levels of miR‐199a‐5p (*t* test, t_(4)_ = −13.350, *p* = .000) (*p* < .01) (Figure 3a), and miR‐199a‐5p‐inhibitor inhibited the expression of miR‐199a‐5p (*t* test, t_(4)_ = 10.521, *p* = .000) (*p* < .01) (Figure 3d). In addition, overexpression of miR‐199a‐5p suppressed cell reproduction of hippocampal neuron (*t* test, t_(4)_ = 9.080, *p* = .001) (*p* < .01) and enhanced neuron’ apoptotic cells (*t* test, t_(4)_ = −11.106, *p* = .000) (*p* < .01) (Figure 3b and 3c). And miR‐199a‐5p‐inhibitor had the reverse effect (Figure 3e, *t* test, t_(4)_ = −6.662, *p* = .003; Figure 3f, *t* test, t_(4)_ = 7.146, *p* = .002) (*p* < .01) (Figure 3e and 3f).

### 3.4 WNT2 was a direct target of MIR‐199a‐5p

**FIGURE 4:**
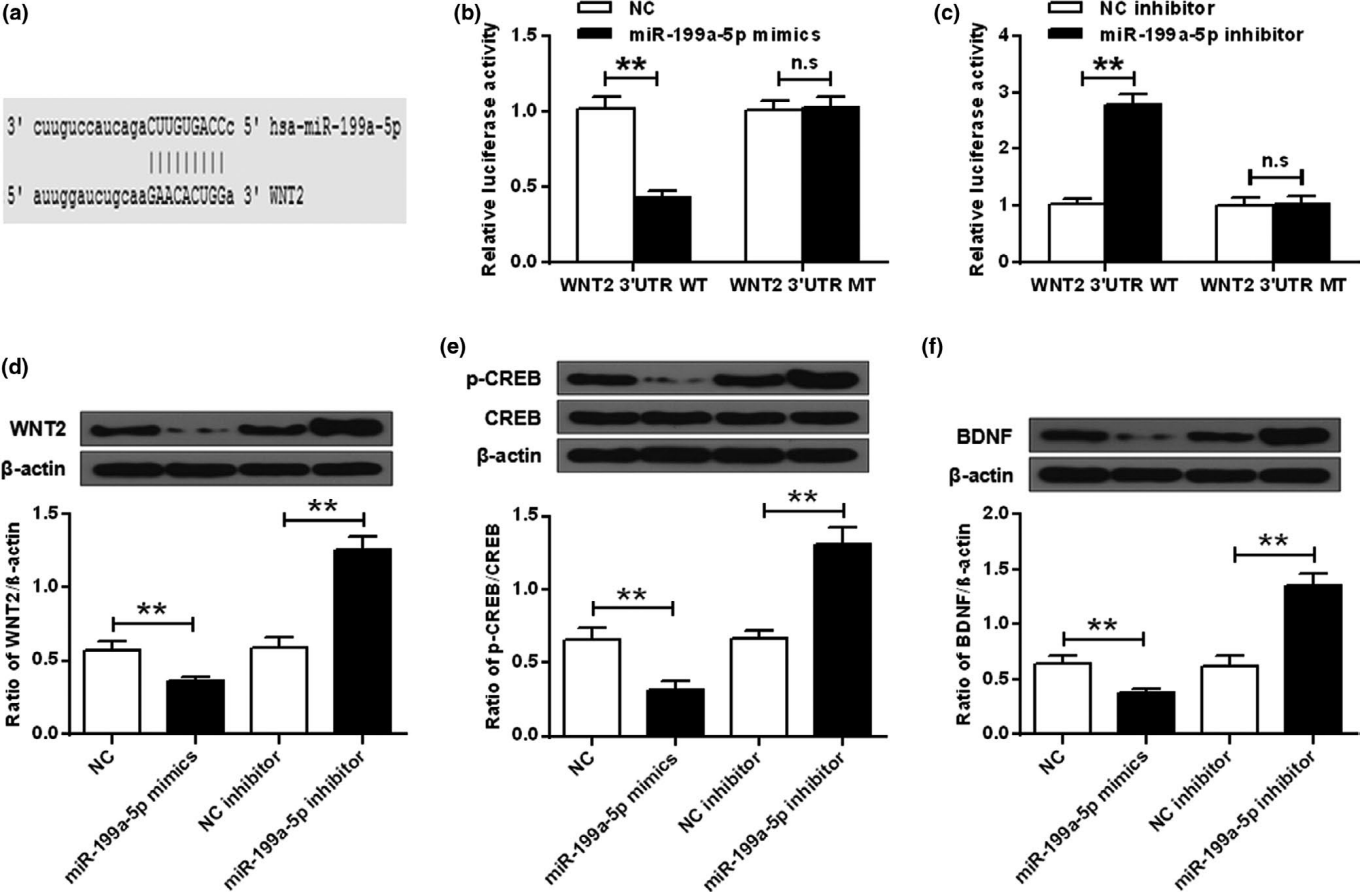
WNT2 was targeted by miR‐199a‐5p. (a) Shared sequences in miR‐199a‐5p and WNT2. (b) Luciferase results of WNT2‐WT for cell in transfection with miR‐199a‐5p‐mimic. (c) Luciferase results of WNT2‐WT for cell in transfection with miR‐199a‐5p‐inhibitor. Hippocampal neuron in transfection with miR‐199a‐5p mimics/inhibitor: (d) The expression of WNT2. (e) The expression of p‐CREB. (f) The expression of BDNF. **p < .01

The possible binding between miR‐199a‐5p and WNT2 was predicted by MicroRNA.org (Figure 4a). Dual‐luciferase reporter assay results demonstrated that the expression levels of WNT2‐WT were significantly reduced in cells with the overexpression of miR‐199a‐5p (ANOVA, *F*_(3,8)_ = 63.339, *p* = .000; LSD test, t = 11.251, *p* = .000) (*p* < .01), while the expression levels of WNT2‐WT were substantially elevated in cells transfected with miR‐199a‐5p‐inhibitor (ANOVA, *F*_(3,8)_ = 103.402, *p* = .000; LSD test, t = −14.353, *p* = .000) (*p* < .01) (Figure 4b and c). In addition, the expression lof WNT2 (ANOVA, *F*_(3,8)_ = 104.709, *p* = .000; LSD test, t = 3.888, *p* = .005) (Figure 4d), p‐CREB (ANOVA, *F*_(3,8)_ = 81.553, *p* = .000; LSD test, t = 5.270, *p* = .001) (Figure 4e) and BDNF (ANOVA, *F*_(3,8)_ = 78.783, *p* = .000; LSD test, t = 3.897, *p* = .005) (Figure 4f) in hippocampal neuron in transfection with miR‐199a‐5p mimics were significantly suppressed (*p* < .01) but significantly elevated with the transfection of miR‐199a‐5p‐inhibitor (Figure 4d, ANOVA, *F*_(3,8)_ = 104.709, *p* = .000; LSD test, t = −12.405, *p* = .000; Figure 4e, ANOVA, *F*_(3,8)_ = 81.553, *p* = .000; LSD test, t = −9.875, *p* = .000; Figure 4f, ANOVA, *F*_(3,8)_ = 78.783, *p* = .000; LSD test, t = −10.943, *p* = .000) (*p* < .01).

### 3.5 Silencing of WNT2 attenuated the effect of MIR‐199a‐5p‐inhibitor on cell reproduction and apoptosis of hippocampal neuron

**FIGURE 5:**
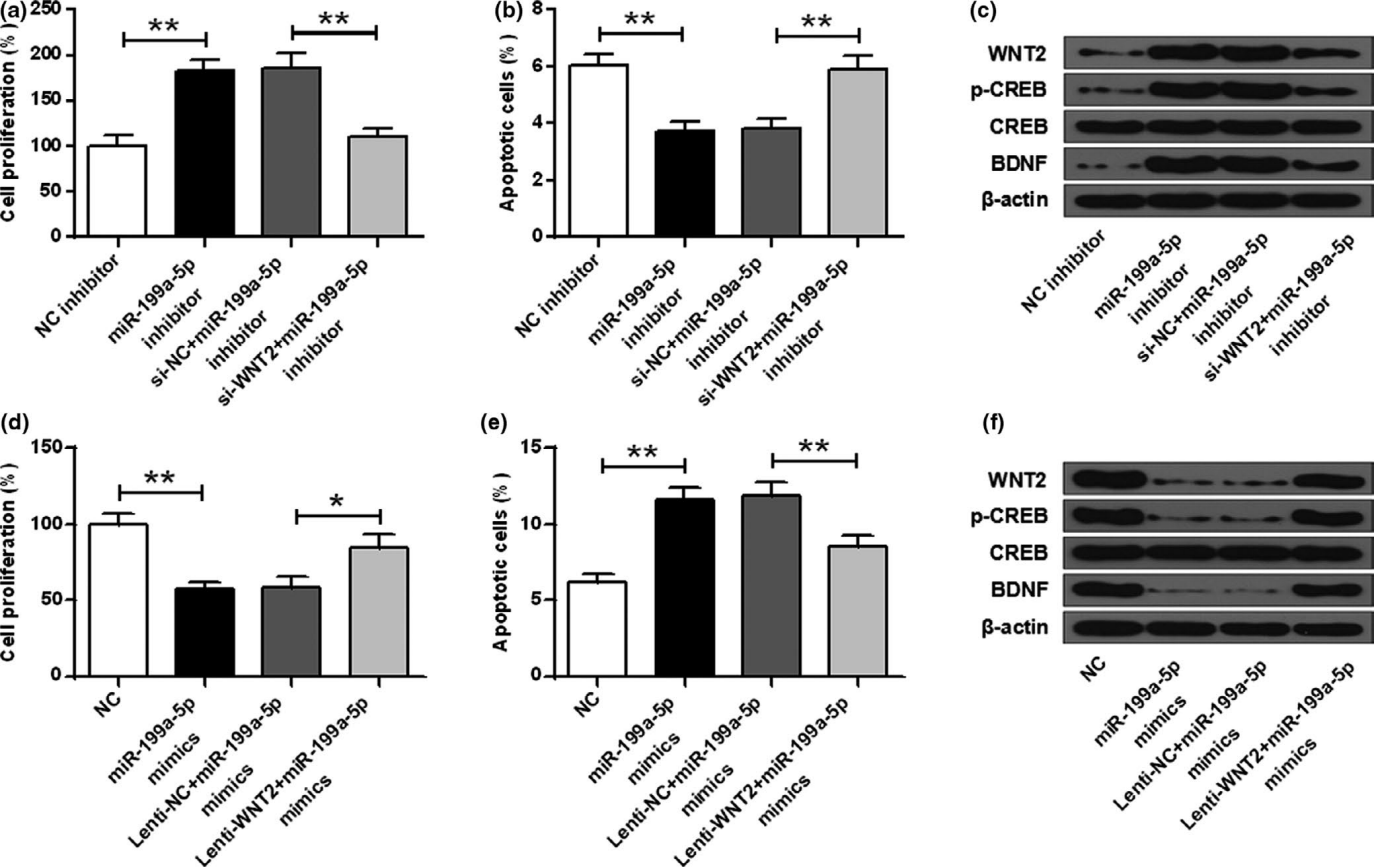
Silencing of WNT2 attenuated the effect of miR‐199a‐5p‐inhibitor on cell reproduction and apoptosis of hippocampal neuron. Hippocampal neuron transfected with NC‐inhibitor, miR‐199a‐5p‐inhibitor, miR‐199a‐5p‐inhibitor + si‐NC, or miR‐199a‐5p‐inhibitor + si‐WNT2: (a) The cell reproduction. (b) Cell apoptosis. (c) Expressions of WNT2, p‐CREB, and BDNF. Hippocampal neuron transfected with NC, miR‐199a‐5p mimics, miR‐199a‐5p mimics + Lenti‐NC, or miR‐199a‐5p mimics + Lenti‐WNT2: (d) The cell reproduction. (e) Cell apoptosis. (f) The expression of WNT2, p‐CREB, and BDNF. * *p* < .05, ** *p* < .01

As shown in Figure 5a, the miR‐199a‐5p‐inhibitor could enhance cell reproduction of hippocampal neuron (ANOVA, *F*_(3,8)_ = 41.025, *p* = .000; LSD test, t = −8.137, *p* = .000), but si‐WNT2 attenuated the effect (ANOVA, *F*_(3,8)_ = 41.025, *p* = .000; LSD test, t = 7.476, *p* = .000) (*p* < .01). In addition, si‐WNT2 relieved the inhibition effect from miR‐199a‐5p‐inhibitor on the apoptosis of neurons (ANOVA, *F*_(3,8)_ = 32.660, *p* = .000; LSD test, t = −6.658, *p* = .000) (*p* < .05) (Figure 5b). Western blotting results showed that miR‐199a‐5p‐inhibitor can elevate the expression levels of WNT2, p‐CREB, and BDNF, and this effect was were reversed by si‐WNT2 (Figure 5c). In addition, miR‐199a‐5p mimic could suppress cell reproduction of hippocampal neuron (ANOVA, *F*_(3,8)_ = 25.246, *p* = .000; LSD test, t = 7.255, *p* = .000) (*p* < .01) (Figure 5d), enhance apoptosis of neuron (ANOVA, *F*_(3,8)_ = 37.176, *p* = .000; LSD test, t = −8.670, *p* = .000) (*p* < .01) (Figure 5e), and inhibit the expression of WNT2, p‐CREB and BDNF (Figure 5f). However, Lenti‐WNT2 attenuated these effects (Figure 5d, ANOVA, *F*_(3,8)_ = 25.246, *p* = .000; LSD test, t = −4.019, *p* = .016; Figure 5e, ANOVA, *F*_(3,8)_ = 37.176, *p* = .000; LSD test, t = 5.299, *p* = .001) (*p* < .05, *p* < .01).

### 3.6 MIR‐199a‐5p‐inhibitor relived the depressive‐like symtomin cums mice

**FIGURE 6:**
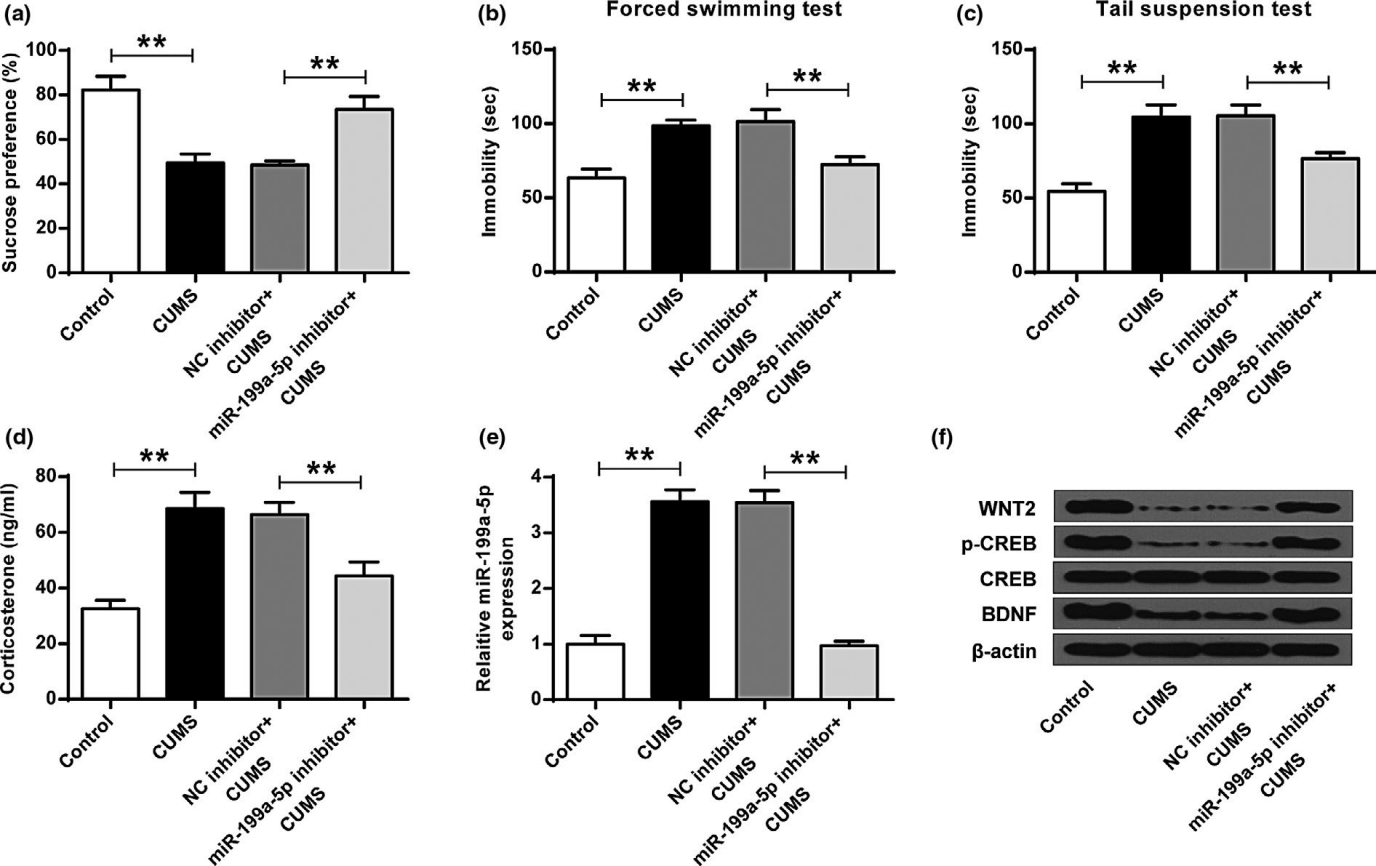
miR‐199a‐5p‐inhibitor relived depressive‐like CUMS mice. In the control, CUMS, CUMS + NC‐inhibitor, and CUMS + miR‐199a‐5p‐inhibitor: (a) sucrose preference test. (b) forced swimming test. (c) tail suspension test. (d) The contents of corticosterone. (e) The expression of miR‐199a‐5p. (f) The expression of WNT2, p‐CREB, and BDNF. *p < .05, ** *p* < .01

As shown in Figure 6a, miR‐199a‐5p‐inhibitor changed the impact of CUMS on sucrose preference (SP) (ANOVA, *F*_(3,20)_ = 38.436, *p* = .000; LSD test, t = −6.399, *p* = .000) as well as the immobility duration in forced swimming test (ANOVA, *F*_(3,20)_ = 29.918, *p* = .000; LSD test, t = 5.925, *p* = .000) (Figure 6b) and tail suspension test (ANOVA, *F*_(3,20)_ = 45.154, *p* = .000; LSD test, t = 5.614, *p* = .001) (Figure 6c) (*p* < .01). Moreover, miR‐199a‐5p‐inhibitor reversed the increasing in the expression levels of CORT (ANOVA, *F*_(3,20)_ = 42.610, *p* = .000; LSD test, t = 5.825, *p* = .000) (*p* < .05) (Figure 6d) and miR‐199a‐5p (ANOVA, *F*_(3,20)_ = 216.284, *p* = .000; LSD test, t = 18.058, *p* = .000) (*p* < .01) (Figure 6e), and the decreasing in the expression levels of WNT2, p‐CREB and BDNF (Figure 6f) resulted from CUMS.

### 3.7 Overexpression of MIR‐199a‐5p attenuated the effect of fluoxetine on cums mice

**FIGURE 7:**
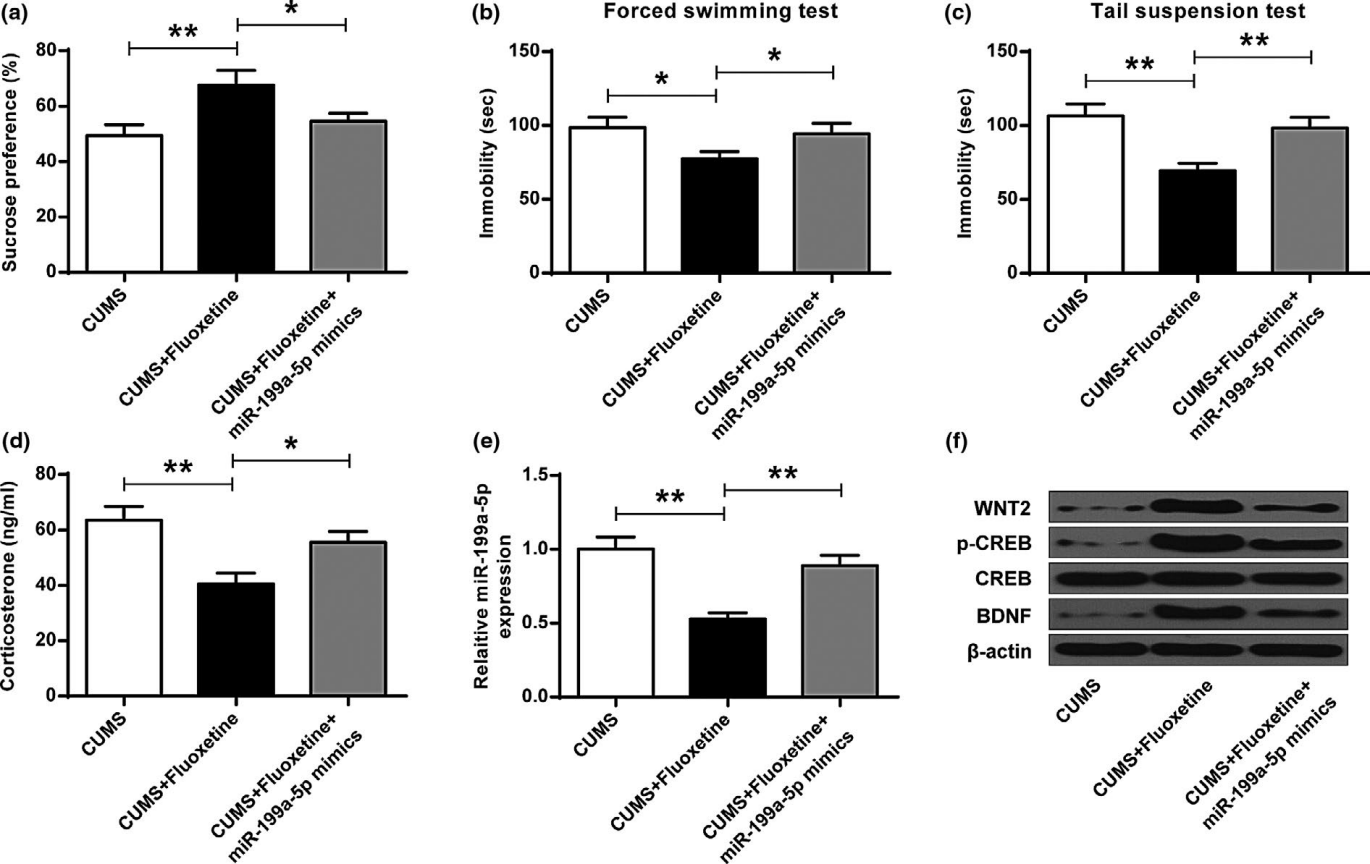
Overexpression of miR‐199a‐5p reversed the effect of fluoxetine on CUMS mice. In CUMS, CUMS + fluoxetine, and CUMS + fluoxetine +miR‐199a‐5p mimic: (a) sucrose preference test. (b) forced swimming test. (c) tail suspension test. (d) CORT concentrations. (e) The expression of miR‐199a‐5p. (f) The expression of WNT2, p‐CREB, and BDNF. * *p* < .05, ** *p* < .01

It showed that fluoxetine could elevate the SP of CUMS mice (ANOVA, *F*_(2,15)_ = 15.122, *p* = .000; LSD test, t = −5.333, *p* = .002) (Figure 7a), decrease the immobility duration in forced swimming test (ANOVA, *F*_(2,15)_ = 9.321, *p* = .000; LSD test, t = 4.289, *p* = .013) (Figure 7b) and tail suspension test (ANOVA, *F*_(2,15)_ = 23.699, *p* = .001; LSD test, t = 6.541, *p* = .001) (Figure 7c) (*p* < .05, *p* < .01). However, overexpression of miR‐199a‐5p attenuated this effect (Figure 7a, ANOVA, *F*_(2,15)_ = 15.122, *p* = .000; LSD test, t = 3.725, *p* = .020; Figure 7b, ANOVA, *F*_(2,15)_ = 9.321, *p* = .000; LSD test, t = −3.275, *p* = .017; Figure 7c, ANOVA, *F*_(2,15)_ = 23.699, *p* = .001; LSD test, t = −5.131, *p* = .002) (*p* < .05). In addition, the decreasing in the expression levels of CORT (ANOVA, *F*_(2,15)_ = 21.792, *p* = .002; LSD test, t = 6.500, *p* = .001) (*p* < .01) (Figure 7d) and miR‐199a‐5p (ANOVA, *F*_(2,15)_ = 42.179, *p* = .000; LSD test, t = 8.795, *p* = .000) (*p* < .01) (Figure 7e), and the increasing in the expression levels of WNT2, p‐CREB and BDNF (Figure 7f) resulted from fluoxetine, were all reversed by miR‐199a‐5p mimic (Figure 7d, ANOVA, *F*_(2,15)_ = 21.792, *p* = .002; LSD test, t = −4.648, *p* = .010; Figure 7e, ANOVA, *F*_(2,15)_ = 42.179, *p* = .000; LSD test, t = −6.689, *p* = .001) (*p* < .05, *p* < .01).

## 4 DISCUSSION

Altered expression of miRNAs in blood has been reported in depressed patients during antidepressant treatments (Bocchio‐Chiavetto et al., 2013). Besides, altered expression of miRNA network in locus coeruleus of depressed suicide subjects were also reported (Roy et al., 2017). In this study, the expression of candidate miRNAs in CSF and serum in MDD patient were investigated. It was found that the expression levels of miR‐139‐5p and miR‐199a‐5p were elevated in CSF and MDD serum, and the increase in the expression levels of miR‐199a‐5p were more obvious compared with the control patient. To the best of our knowledge, we are the first to discover that miR‐199a‐5p was up‐regulated in depressive samples.

It was reported that the expression levels of miR‐132 were significantly increased in patients with depression (Fang et al., 2018). CUMS mice were shown to have increased depression‐like behaviors and reduced hippocampal expression levels of miR‐124 (Higuchi et al., 2016). In our study, the expression levels of miR‐199a‐5p in CUMS mice were elevated compared to that in the control mouse. The expression levels of WNT2, p‐CREB, and BDNF in CUMS mice were reduced. In addition, we also observed a considerable reduction of sucrose preference in CUMS. The concentrations of CORT in CUMS serum were enhanced. Similar to previous studies, we found that the expression levels of miR‐199a‐5p were substantially elevated in the hippocampus of CUMS mice.

MiRNAs were identified to involve in numeric biological functions, including developmental transitions (La Torre et al., 2013), neuronal patterning (Kosik, 2006), cell apoptosis (Cheng et al., 2005), and fat metabolism (P. Xu et al., 2003). miR‐199a‐5p was reported to be involved in the promotion of cell reproduction in autosomal dominant polycystic kidney diseases (L. Sun, Zhu, et al., 2015). However, in our study, we noticed that overexpression of miR‐199a‐5p suppressed cell reproduction and enhanced apoptosis of hippocampal neuron.

It was reported that miR‐199a‐5p could target the WNT2 signaling pathway and regulate cell reproduction of smooth muscle cells (Gheinani et al., 2015). Similarly, we also found possible binding between miR‐199a‐5p and WNT2. The expression levels of WNT2‐WT were significantly reduced in cells with the overexpression of miR‐199a‐5p, while the activities of WNT2‐WT were considerably elevated in cells transfected with miR‐199a‐5p‐inhibitor. In addition, the expression of WNT2, p‐CREB, and BDNF in hippocampal neuron transfected by miR‐199a‐5p mimics were suppressed. Knockdown of WNT2 attenuated the effects of miR‐199a‐5p‐inhibitor on cell reproduction and apoptosis of hippocampal neuron. These data further confirmed that WNT2 was a direct target of miR‐199a‐5p.

MiR‐124 was reported to be the most abundant in the brain, and its dysregulation has been related to neurodegeneration, neuroimmune disorder, and CNS stress (Sun et al., 2015). In this study, we found that miR‐199a‐5p could act as a novel marker for major depression‐related brain neuronal regulations. Our data showed that miR‐199a‐5p‐inhibitor changed the effect of CUMS on SP, as well as the immobility duration in the forced swimming test and tail suspension test. Moreover, miR‐199a‐5p‐inhibitor reversed the increasing of the expression levels of CORT and miR‐199a‐5p, and the decreasing of the expression levels of WNT2, p‐CREB, and BDNF resulted from CUMS.

Fluoxetine could increase the activity of the ERK‐CREB signal system and alleviates the depressive‐like behaviors in rats exposed to chronic forced swim stress (Qi et al., 2008). In this study, we also found that fluoxetine can elevate the SP of CUMS mice and decrease the immobility duration in the forced swimming test and tail suspension test. However, overexpression of miR‐199a‐5p relieved the effect. In addition, the decreasing in the expression levels of CORT and miR‐199a‐5p, and the increasing in the expression levels of WNT2, p‐CREB, and BDNF resulted from fluoxetine, were all reversed by a miR‐199a‐5p mimic. It indicated that miR‐199a‐5p had the opposite function of fluoxetine on CUMS mice.

## 5 CONCLUSIONS

MiR‐199a‐5p can target WNT2 to enhance the developments of depression by regulating the CREB/BDNF signaling.

## CONFLICT OF INTEREST

The authors declare that they have no conflict of interest.

## AUTHOR CONTRIBUTION

Jianli Yang designed the study. Zheng Liu carried out experiments and wrote the manuscript, Jianli Yang revised the paper, Qing Fang, Hua Shao, Dalu Yang, Junfang Sun and Lizhi Gao collected patient specimens and related information. Jianli Yang, Qing Fang, Hua Shao, Dalu Yang, Junfang Sun and Lizhi Gao contributed to analyzing the data. All authors reviewed the results and approved the final version of the manuscript.

### PEER REVIEW

The peer review history for this article is available at https://publons.com/publon/10.1002/brb3.2107

## Data Availability

The analyzed data sets generated during the study are available from the corresponding author on reasonable request.
